# Supplementary material for: The proportion of randomized controlled trials that inform clinical practice
Source: eLife. 2022 Aug 17;11:e79491. doi: 10.7554/eLife.79491 (PMC9427100; doi:10.7554/eLife.79491)
Supplement: Supplementary file 1. [file elife-79491-supp1.docx]

**Supplementary File 1 – Index for Supplementary Files**

Supplementary File 1 – Index

Supplementary File 2 – Proportion of Trials Meeting Each Criterion for Informativeness

Supplementary File 3 – Trials Not Fulfilling Feasibility Condition

Supplementary File 4 – Trials Not Reported

Supplementary File 5 – Trials Not Cited in Clinical Review Documents

Supplementary File 6 – Trials with Concerns Regarding Design

Figure 2 – Figure Supplement 1 – The Cumulative Proportion of Trials Meeting Four Conditions of Informativeness by Sponsor

Supplementary File 7 – Phase 4 Trials Not Meeting 4 Informativeness Criteria

Supplementary File 8 – ClinicalTrials.gov search criteria

Supplementary File 9 – Trial Inclusion and Exclusion criteria

Figure 3 – Figure Supplement 1 – Flow Diagram for Ischemic Heart Disease Interventional Trials.pdf

Figure 3 – Figure Supplement 2 – Flow Diagram for Diabetes Mellitus Interventional Trials

Figure 3 – Figure Supplement 3 – Flow Diagram for Lung Cancer Interventional Trials

Supplementary File 10 – Assessment of Regulatory Approval Status

Supplementary File 11 – Addressing 4 Conditions for Informative Clinical Trials

Supplementary File 12 – Classification of Reason for Termination

Supplementary File 13 – Methodology for Publication Search

Supplementary File 14 – Systematic Review Citation Search Strategy and Quality Assessment

Supplementary File 15 – Clinical Practice Guideline and Point-of-Care Medical Database Search Strategies and Quality Assessment

Supplementary File 16 – Operationalization of modified Cochrane Risk of Bias score

Supplementary File 17 – Inter-rater Agreement Rates

Supplementary File 18 – Deviations to the Study Protocol

Supplementary File 19 – STROBE Checklist for Cohort Studies
