# Supplementary material for: The proportion of randomized controlled trials that inform clinical practice
Source: eLife. 2022 Aug 17;11:e79491. doi: 10.7554/eLife.79491 (PMC9427100; doi:10.7554/eLife.79491)
Supplement: Supplementary file 2. [file elife-79491-supp2.docx]

**Supplementary File 2 – Proportion of Trials Meeting Each Criterion for Informativeness**

| **Condition for Informativeness** | **Ratio** | **% (95% CI)** |
| --- | --- | --- |
| Feasibility | 90 of 125 trials | 72.0 (63.3 – 79.7) |
| Reporting | 81 of 90 trials | 90.0 (81.9 – 95.3) |
| Importance | 63 of 81 trials | 77.8 (67.2 – 86.3) |
| Design | 33 of 63 trials | 52.4 (39.4 – 65.1) |
