# Supplementary material for: The proportion of randomized controlled trials that inform clinical practice
Source: eLife. 2022 Aug 17;11:e79491. doi: 10.7554/eLife.79491 (PMC9427100; doi:10.7554/eLife.79491)
Supplement: Supplementary file 3. — IHD: ischemic heart disease; DM: diabetes mellitus; lung CA: lung cancer; PCD: primary completion date. [file elife-79491-supp3.docx]

**Supplementary File 3 –** **Trials Not Fulfilling Feasibility Condition**

| **NCT** | **Disease** | **Phase** | **Trial Status** | **Feasibility Criteria** |
| --- | --- | --- | --- | --- |
| NCT00794664 | IHD | 3 | Completed | <85% enrollment |
| NCT00860847 | IHD | 3 | Completed | <85% enrollment |
| NCT00901277 | IHD | NA | Completed | <85% enrollment |
| NCT00965055 | IHD | 3 | Terminated | Uninformative Termination |
| NCT01106534 | IHD | 4 | Completed | <85% enrollment |
| NCT01221272 | IHD | 4 | Completed | <85% enrollment |
| NCT01078272 | IHD | NA | Terminated | Uninformative Termination |
| NCT01246011 | IHD | 4 | Terminated | Uninformative Termination |
| NCT01177592 | IHD | NA | Terminated | Uninformative Termination |
| NCT01205776 | IHD | NA | Completed | <85% enrollment |
| NCT01213251 | IHD | 2 | Completed | <85% enrollment |
| NCT01231750 | IHD | 3 | Terminated | Uninformative Termination |
| NCT01427218 | IHD | NA | Unknown  (Recruiting) | 6.0 x the planned PCD timeline |
| NCT01810029 | IHD | 2 | Unknown  (Active, Not Recruiting) | 3.4 x the planned PCD timeline |
| NCT00608387 | DM | NA | Completed | <85% enrollment |
| NCT00802152 | DM | NA | Completed | <85% enrollment |
| NCT00812253 | DM | 2 | Completed | <85% enrollment |
| NCT00869362 | DM | NA | Completed | <85% enrollment |
| NCT00889785 | DM | NA | Completed | <85% enrollment |
| NCT01017523 | DM | 3 | Completed | <85% enrollment |
| NCT01098253 | DM | NA | Completed | <85% enrollment |
| NCT00939250 | DM | 4 | Completed | <85% enrollment |
| NCT01136785 | DM | NA | Completed | <85% enrollment |
| NCT01107717 | DM | 4 | Active, Not Recruiting | 4.4 x the planned PCD timeline |
| NCT01267448 | DM | 4 | Unknown (Recruiting) | 5.4 x the planned PCD timeline |
| NCT01324921 | DM | 2/3 | Completed | <85% enrollment |
| NCT00863746 | Lung CA | 3 | Completed | <85% enrollment |
| NCT00887315 | Lung CA | 2 | Terminated | Uninformative Termination |
| NCT00932152 | Lung CA | 2 | Terminated | Uninformative Termination |
| NCT00982111 | Lung CA | 3 | Completed | <85% enrollment |
| NCT01012401 | Lung CA | NA | Completed | <85% enrollment |
| NCT01055197 | Lung CA | 2 | Completed | <85% enrollment |
| NCT01107626 | Lung CA | 3 | Active, Not Recruiting | 5.2 x the planned PCD timeline |
| NCT01179308 | Lung CA | NA | Terminated | Uninformative Termination |
| NCT01413750 | Lung CA | 1/2 | Terminated | Uninformative Termination |

IHD – Ischemic Heart Disease

DM – Diabetes Mellitus

Lung CA – Lung Cancer

PCD – Primary Completion Date
