# Supplementary material for: The proportion of randomized controlled trials that inform clinical practice
Source: eLife. 2022 Aug 17;11:e79491. doi: 10.7554/eLife.79491 (PMC9427100; doi:10.7554/eLife.79491)
Supplement: Supplementary file 4. — IHD: ischemic heart disease; DM: diabetes mellitus; lung CA: lung cancer. [file elife-79491-supp4.docx]

**Supplementary File 4 –** **Trials Not Reported**

| **NCT** | **Disease** | **Phase** | **Trial Status** | **Why Not Reported** |
| --- | --- | --- | --- | --- |
| NCT00888537 | IHD | NA | Completed | Exempt from FDAAA 801: not evaluating a drug, biologic or device products |
| NCT01050348 | IHD | 4 | Completed | Subject to FDAAA 801: unclear why it is not reported. |
| NCT01141192 | DM | NA | Completed | Exempt from FDAAA 801: not evaluating a drug, biologic or device products |
| NCT00935441 | DM | NA | Completed | Exempt from FDAAA 801: not evaluating a drug, biologic or device products |
| NCT01067950 | DM | 3 | Unknown (Recruiting) | Not yet subject to FDAAA 801 reporting requirements as trial has not reached primary completion date. |
| NCT00950677 | DM | 4 | Completed | Subject to FDAAA 801: Results submitted to ClinicalTrials.gov December 4, 2020. Results returned to investigator December 30, 2020 after quality control review. No evidence of results re-submission. |
| NCT01197963 | DM | NA | Terminated | Exempt from FDAAA 801: not evaluating a drug, biologic or device products |
| NCT01209312 | DM | NA | Completed | Exempt from FDAAA 801: not evaluating a drug, biologic or device products |
| NCT00863512 | Lung  CA | 3 | Terminated | Subject to FDAAA 801: As per the trial registration record: “Study terminated prematurely with 34 participants recruited. Per protocol, 338 events were needed to conduct the primary analysis; therefore, the planned analyses was not performed due to a lack of events and a termination of data collection.” |

IHD – Ischemic Heart Disease

DM – Diabetes Mellitus

Lung CA – Lung Cancer
