# Supplementary material for: The proportion of randomized controlled trials that inform clinical practice
Source: eLife. 2022 Aug 17;11:e79491. doi: 10.7554/eLife.79491 (PMC9427100; doi:10.7554/eLife.79491)
Supplement: Supplementary file 5. — IHD: ischemic heart disease; DM: diabetes mellitus; lung CA: lung cancer. [file elife-79491-supp5.docx]

**Supplementary File 5 – Trials Not Cited in Clinical Review Documents**

| **NCT** | **Disease** | **Title** | **Phase** | **Trial Status** | **Sponsor** | **Published Results** |
| --- | --- | --- | --- | --- | --- | --- |
| NCT00924118 | IHD | A Safety and Efficacy Evaluation of Sodium Nitrite Injection for the Prevention of Ischemia-Reperfusion Injury Associated with Acute Myocardial Infarction | 2 | Completed | Non-Industry | NO |
| NCT00954707 | IHD | A Prospective, Randomized, Multi-Center, Double-Blind Trial to Assess the Effectiveness and Safety of Different Durations of Dual Anti-Platelet Therapy (DAPT) in Subjects Undergoing Percutaneous Coronary Intervention with the CYPHER Sirolimus-eluting Coronary Stent | 4 | Unknown (Previously, Active, Not Recruiting) | Industry | NO |
| NCT01175018 | IHD | Anakinra to Prevent Adverse Post-infarction Remodeling | 2 | Completed | Non-Industry | YES |
| NCT01178268 | IHD | XIENCE V Everolimus Eluting Coronary Stent System China: Post-Approval Randomized Control Trial | 4 | Completed | Industry | NO |
| NCT01230892 | IHD | The Evaluation of The Effects of Nebivolol in Comparison to Atenolol on Wall Shear Stress and Rupture Prone Coronary Artery Plaques in Patients With Moderate Coronary Artery Disease | 4 | Completed | Non-Industry | YES |
| NCT01101867 | DM | Prandial Insulin Dosing Using the Carbohydrate Counting Technique in Hospitalized Patients With Diabetes | 4 | Completed | Non-Industry | YES |
| NCT01194245 | DM | A Phase II, Randomized, Double Blind, 2-Way Crossover Safety and Efficacy Study of Subcutaneously Injected Prandial Insulins: Lispro-PH20 or Aspart-PH20 Compared to Insulin Lispro (Humalog) in Patients With Type 1 Diabetes | 2 | Completed | Industry | NO |
| NCT00916357 | DM | Phase 2, Double-Blind Randomized, 3-way Cross-Over Liquid Meal Study With Optimal Doses of SC Administered Insulin Lispro With and Without rHuPH20 and Regular Human Insulin With rHuPH20 to Compare Pharmacokinetics, Postprandial Glycemic Response, and Optimal Insulin Dose in Patients With T2DM | 2 | Completed | Industry | YES |
| NCT00993824 | DM | Use of Continuous Glucose Monitoring With Ambulatory Glucose Profile Analysis to Demonstrate the Glycemic Effect of Colesevelam HCl (Welchol) in Patients With Type 2 Diabetes | 2/3 | Completed | Non-Industry | YES |
| NCT00918138 | DM | A 4-Week, Multicenter, Randomized, Double-Blind, Phase 3b Trial to Evaluate the Efficacy of Saxagliptin in Combination With Metformin XR 1500 mg Versus Up-titrated Metformin XR to 2000 mg in Subjects With Type 2 Diabetes Who Have Inadequate Glycemic Control With Diet and Exercise and a Stable Dose of Metformin XR 1500 mg | 3 | Completed | Industry | YES |
| NCT00844090 | DM | The Role of Apathy in Glycemic Control | NA | Completed | Non-Industry | NO |
| NCT00978796 | DM | Pilot Study Assessing Glucose Effects of Sitagliptin (Januvia) in Adult Patients With Type 1 Diabetes | 4 | Completed | Non-Industry | YES |
| NCT01089569 | DM | Evaluation of Insulin Glargine and Exenatide: A Randomized Clinical Trial with Continuous Glucose Monitoring and Ambulatory Glucose Profile Analysis | NA | Completed | Non-Industry | YES |
| NCT00918203 | Lung CA | A Randomized Phase 2 Study of Human Anti-PDGFRa Monoclonal Antibody (IMC-3G3) with Paclitaxel/Carboplatin or Paclitaxel/Carboplatin Alone in Previously Untreated Patients with Locally Advanced or Metastatic Non-Small Cell Lung Cancer | 2 | Completed | Industry | YES |
| NCT00976677 | Lung CA | Randomized Double-Blind Placebo Controlled Phase II Trial Evaluating Erlotinib in Non-Smoking Patients With (Bevacizumab-Eligible and Ineligible) Advanced Non-Small Cell Lung Cancer | 2 | Terminated | Non-Industry | NO |
| NCT00977470 | Lung CA | Phase II Study of Erlotinib With or Without Hydroxychloroquine in Patients With Previously Untreated Advanced NSCLC and EGFR Mutations | 2 | Active, NR | Non-Industry | NO |
| NCT01085136 | Lung CA | Phase III Randomized Trial of BIBW 2992 Plus Weekly Paclitaxel Versus Investigator’s Choice of Chemotherapy Following BIBW 2992 Monotherapy in Non-Small Cell Lung Cancer Patients Failing Previous Erlotinib or Gefitinib Treatment (LUX Lung 5) | 3 | Completed | Industry | YES |
| NCT01104155 | Lung CA | Eribulin Mesylate in Combination with Intermittent Erlotinib in Patients with Previously Treated, Advanced Non-Small Cell Lung Cancer | 2 | Completed | Industry | YES |

IHD – Ischemic Heart Disease

DM – Diabetes Mellitus

Lung CA – Lung Cancer
