# Supplementary material for: The proportion of randomized controlled trials that inform clinical practice
Source: eLife. 2022 Aug 17;11:e79491. doi: 10.7554/eLife.79491 (PMC9427100; doi:10.7554/eLife.79491)
Supplement: Supplementary file 6. — IHD: ischemic heart disease; DM: diabetes mellitus; lung CA: lung cancer. [file elife-79491-supp6.docx]

**Supplementary File 6 – Trials with Concerns Regarding Design**

| **NCT** | **Disease** | **Phase** | **Trial Status** | **High Risk Elements^a^** | **Extracted** |
| --- | --- | --- | --- | --- | --- |
| NCT00823212 | IHD | 3 | Completed | Blinding of Participants & Personnel  Blinding of Outcome Assessment | No |
| NCT00977938 | IHD | 4 | Completed | Blinding of Outcome Assessment | Yes |
| NCT01069003 | IHD | 4 | Completed | Selective Reporting | No |
| NCT01033916 | IHD | NA | Unknown  (Active, Not Recruiting) | Blinding of Participants & Personnel | Yes |
| NCT01086800 | IHD | 2 | Completed | Selective Reporting | No |
| NCT01206062 | IHD | NA | Completed | Blinding of Participants & Personnel | Yes |
| NCT01624727 | IHD | NA | Completed | Blinding of Participants & Personnel  Incomplete Outcome Data  Selective Reporting | Yes |
| NCT02357212 | IHD | NA | Completed | Selective Reporting | No |
| NCT00838916 | DM | 3 | Completed | Blinding of Participants & Personnel  Blinding of Outcome Assessment | Yes |
| NCT00856284 | DM | 3 | Completed | Incomplete Outcome Data | Yes |
| NCT00976391 | DM | 3 | Completed | Blinding of Participants & Personnel  Blinding of Outcome Assessment | Yes |
| NCT00997178 | DM | 3 | Completed | Blinding of Participants & Personnel  Blinding of Outcome Assessment | Yes |
| NCT01040676 | DM | NA | Completed | Incomplete Outcome Data | Yes |
| NCT01045447 | DM | 3 | Completed | Blinding of Participants & Personnel | No |
| NCT01073566 | DM | 2 | Completed | Three elements were of Unclear risk of bias | No |
| NCT01189890 | DM | 3 | Completed | Selective Reporting | No |
| NCT00979628 | DM | 4 | Completed | Blinding of Participants & Personnel  Blinding of Outcome Assessment | Yes |
| NCT01222429 | DM | NA | Completed | Selective Reporting | No |
| NCT01231984 | DM | NA | Completed | Blinding of Participants & Personnel | No |
| NCT01221090 | DM | 4 | Completed | Incomplete Outcome Data  Selective Reporting | Yes |
| NCT01264796 | DM | NA | Completed | Selective Reporting | No |
| NCT00768755 | Lung  CA | 1/2 | Completed | Blinding of Participants & Personnel  Blinding of Outcome Assessment  Incomplete Outcome Data | Yes |
| NCT00828139 | Lung  CA | 2 | Completed | Blinding of Participants & Personnel | No |
| NCT00892710 | Lung  CA | 2 | Completed | Four elements were of Unclear risk of bias | No |
| NCT00932893 | Lung  CA | 3 | Completed | Blinding of Participants & Personnel  Blinding of Outcome Assessment | Yes |
| NCT00946712 | Lung  CA | 3 | Active, Not Recruiting | Blinding of Outcome Assessment | No |
| NCT00948675 | Lung  CA | 3 | Completed | Blinding of Participants & Personnel | Yes |
| NCT01027598 | Lung  CA | 2 | Completed | Incomplete Outcome Data | Yes |
| NCT01041781 | Lung  CA | 3 | Terminated | Selective Reporting | Yes |
| NCT01160744 | Lung  CA | 2 | Completed | Blinding of Outcome Assessment | No |

IHD – Ischemic Heart Disease

DM – Diabetes Mellitus

Lung CA – Lung Cancer
