# Supplementary material for: The proportion of randomized controlled trials that inform clinical practice
Source: eLife. 2022 Aug 17;11:e79491. doi: 10.7554/eLife.79491 (PMC9427100; doi:10.7554/eLife.79491)
Supplement: Supplementary file 7. — IHD: ischemic heart disease; DM: diabetes mellitus. [file elife-79491-supp7.docx]

**Supplementary File 7 – Phase 4 Trials Not Meeting All 4 Informativeness Criteria**

| **NCT** | **Disease** | **Title** | **Trial Status** | **Sponsor** | **Informativeness Assessment** |
| --- | --- | --- | --- | --- | --- |
| NCT00954707 | IHD | A Prospective, Randomized, Multi-Center, Double-Blind Trial to Assess the Effectiveness and Safety of Different Durations of Dual Anti-Platelet Therapy in Subjects Undergoing Percutaneous Coronary Intervention With the CYPHER® Sirolimus-eluting Coronary Stent (CYPHER® Stent) | Unknown (Previously, Active, NR) | Industry | Not cited in review documents |
| NCT00977938 | IHD | A Prospective, Multi-center, Randomized, Double-blind Trial to Assess the Effectiveness and Safety of 12 Versus 30 Months of Dual Antiplatelet Therapy in Subjects Undergoing Percutaneous Coronary Intervention With Either Drug-eluting Stent or Bare Metal Stent Placement for the Treatment of Coronary Artery Lesions | Completed | Non-Industry | Elevated Risk of Bias Score |
| NCT01050348 | IHD | A Double Blinded Randomized Placebo Controlled Study: To Investigate the Role of Upstream High Dose Statin Treatment in Patients With ST Segment Elevation Myocardial Infarction | Completed | Non-Industry | Not Reported |
| NCT01069003 | IHD | EDUCATE: a Prospective, Multi-center Study Designed to Collect Real-world Safety and Clinical Outcomes in Subjects Receiving One or More Endeavor Zotarolimus-Eluting Stents and Either Clopidogrel and Aspirin or Prasugrel and Aspirin as Part of a Dual Antiplatelet Therapy Drug Regimen | Completed | Industry | Elevated Risk of Bias Score |
| NCT01106534 | IHD | XIENCE V® Everolimus Eluting Coronary Stent System USA Post- Approval Study (XIENCE V® USA DAPT Cohort) (XVU-AV DAPT) | Completed | Industry | Poor Feasibility |
| NCT01178268 | IHD | XIENCE V Everolimus Eluting Coronary Stent System China: Post-Approval Randomized Control Trial | Completed | Industry | Not cited in review documents |
| NCT01221272 | IHD | A Phase 4, Randomized, Double-Blind, Placebo-Controlled, Cross-over Trial to Evaluate the Effects of Ranolazine on Myocardial Perfusion Assessed by Serial Quantitative Exercise SPECT Imaging | Completed | Industry | Poor Feasibility |
| NCT01230892 | IHD | The Evaluation of The Effects of Nebivolol in Comparison to Atenolol on Wall Shear Stress and Rupture Prone Coronary Artery Plaques in Patients With Moderate Coronary Artery Disease | Completed | Non-Industry | Not cited in review documents |
| NCT01246011 | IHD | Significance of Antibodies to Heparin/Platelet Factor 4 Complex in Vein Graft Patency and Potential Role of Argatroban for Prevention of Vein Graft Occlusion | Terminated | Non-Industry | Poor Feasibility |
| NCT01101867 | DM | Prandial Insulin Dosing Using the Carbohydrate Counting Technique in Hospitalized Patients With Diabetes | Completed | Non-Industry | Not cited in review documents |
| NCT01267448 | DM | A Pilot Study of Outpatient Discharge Therapy With Saxagliptin + Metformin XR or Sulphonylurea for Recently Diagnosed Type 2 Diabetes Presenting With Severe Hyperglycemia | Unknown  (Previously, Recruiting) | Non-Industry | Poor Feasibility |
| NCT00978796 | DM | Pilot Study Assessing Glucose Effects of Sitagliptin (Januvia) in Adult Patients With Type 1 Diabetes | Completed | Non-Industry | Not cited in review documents |
| NCT00979628 | DM | Basal Bolus Versus Basal Insulin Regimen for the Treatment of Hospitalized Patients With Type 2 Diabetes Mellitus | Completed | Non-Industry | Elevated Risk of Bias Score |
| NCT01107717 | DM | Durability of Early Initial Combination Therapy With Exenatide/Pioglitazone/Metformin vs Conventional Therapy in New Onset Type 2 Diabetes | Active, NR | Non-Industry | Poor Feasibility |
| NCT00939250 | DM | A Comprehensive Intervention for Diabetes and Comorbid Depression in Primary Care | Completed | Non-Industry | Poor Feasibility |
| NCT00950677 | DM | The Effect of the Glucagon Suppressors Pramlintide and Exenatide on Postprandial Glucose Metabolism in Children With Type 2 Diabetes Mellitus | Completed | Non-Industry | Not Reported |
| NCT01221090 | DM | Employing Diabetes Self-Management Models to Reduce Health Disparities in Texas | Completed | Non-Industry | Elevated Risk of Bias Score |

IHD – Ischemic Heart Disease

DM – Diabetes Mellitus
