# Supplementary material for: The proportion of randomized controlled trials that inform clinical practice
Source: eLife. 2022 Aug 17;11:e79491. doi: 10.7554/eLife.79491 (PMC9427100; doi:10.7554/eLife.79491)
Supplement: Supplementary file 8. [file elife-79491-supp8.docx]

**Supplementary File 8 – ClinicalTrials.gov search criteria**

1.Condition or disease search terms:

1. ISCHEMIC HEART DISEASE: coronary artery disease OR coronary disease OR coronary heart disease OR coronary occlusion OR acute coronary syndrome OR myocardial ischemia OR angina pectoris OR angina, stable OR angina, unstable OR myocardial infarction OR ischemic heart disease
2. LUNG CANCER: Lung cancer OR lung neoplasm OR lung carcinoma OR non-small-cell lung cancer OR non-small-cell lung carcinoma OR small cell lung cancer OR small cell lung carcinoma OR lung tumor OR lung tumour
3. DIABETES: diabetes mellitus OR diabetes

2. Study type: “Interventional Studies (Clinical Trials)”

3. Recruitment status: “Recruiting, Completed, Suspended, Terminated, Active not recruiting, enrolling by invitation and unknown status”

4. Study start: 2009-01-01 to 2010-12-31
