# Supplementary material for: The proportion of randomized controlled trials that inform clinical practice
Source: eLife. 2022 Aug 17;11:e79491. doi: 10.7554/eLife.79491 (PMC9427100; doi:10.7554/eLife.79491)
Supplement: Supplementary file 9. [file elife-79491-supp9.docx]

**Supplementary File 9 – Trial Inclusion and Exclusion criteria**

Inclusion criteria:

-Primary outcome = clinical decision-related outcome: including mortality, morbidity, quality of life, functional status, need for further interventions, or an appropriate surrogate measures (for example, ejection fraction, Hgb A1c, or progression free survival in ischemic heart disease, diabetes mellitus and lung cancer respectively)

-Intervention of any type (drug, device, behavioral, surgical or other) directed towards the treatment or prevention of ischemic heart disease/lung cancer/diabetes mellitus (and not side-effects of the disease or complications from disease treatment)

-Trials with a US site

-Randomized trials

-Multi-arm trials

-Trials of interventions subject to FDA regulations and are FDA approved prior to trial start

-Trials of interventions not subject to FDA regulations

-Trials of interventions subject to FDA regulations, FDA approved post trial start, and have at least 5 years of follow-up since FDA approval, to allow for ample time for results incorporation into systematic reviews/clinical practice guidelines/UpToDate

Exclusion criteria:

-Exclusively evaluating safety, diagnostic or screening interventions

-Exclusion of phase 0 or phase 1 trials (given these early phase trials would be unlikely to inform clinical decision-making)

-Exclusion of extension studies with primary aim to enable continued access to drug (and without additional post-marketing surveillance outcomes)

-Indeterminate trials, which are ongoing trials have not surpassed double the allotted time for primary outcome completion (as first stated in the historical clinicaltrials.gov registration record)
