# Supplementary material for: The proportion of randomized controlled trials that inform clinical practice
Source: eLife. 2022 Aug 17;11:e79491. doi: 10.7554/eLife.79491 (PMC9427100; doi:10.7554/eLife.79491)
Supplement: Supplementary file 10. [file elife-79491-supp10.docx]

**Supplementary File 10 – Assessment of Regulatory Approval Status**

Two authors (NH & HM) independently evaluated all eligible trials for regulatory approval status using Drugs@FDA^1^ for drug and biological interventions and the 510(k) Premarket Notification website for devices.^2^ Interventions were classified into one of 3 categories: i) FDA approved prior to trial start (drug, biological or device interventions approved for any use by the time of trial start); ii) FDA approved at least 5 years ago ((drug, biological or device interventions approved for any use prior to October 31, 2016); and, iii) interventions not subject to FDA approval.

Bibliography

1. Drugs@FDA <https://www.accessdata.fda.gov/scripts/cder/daf/index.cfm>.

2. 510(k) Premarket Notification <https://www.accessdata.fda.gov/scripts/cder/daf/index.cfm>.
