# Supplementary material for: The proportion of randomized controlled trials that inform clinical practice
Source: eLife. 2022 Aug 17;11:e79491. doi: 10.7554/eLife.79491 (PMC9427100; doi:10.7554/eLife.79491)
Supplement: Supplementary file 11. — 1Column ‘Conditions for informativeness’ extracted from column 1 in eTable 1 Zarin et al., 2019 [file elife-79491-supp11.docx]

**Supplementary File 11 – Addressing the 4 Conditions for Informative Clinical Trials**

| **Conditions for Informativeness^1^** | **Manner in which this is evaluated** |
| --- | --- |
| Importance:  Trial hypothesis is likely to inform an important scientific, medical or policy question | -Trials are selected for inclusion in our cohort based on their potential to inform clinical decision-making, based on presence of a primary clinical outcome or appropriate surrogate.  -We also assess importance by evaluating the proportion of trials that are cited in clinical review documents (systematic reviews, clinical practice guidelines or point-of-care medical database articles). |
| Design:  Trial methods are likely to provide meaningful evidence related to study hypothesis | -We assess trials that are designed to inform clinical decision-making for evidence of low risk of bias |
| Feasibility:  Trial is likely to be feasible | -Feasible trials include:  -Completed trials that have reached ≥ 85% planned recruitment  -Terminated trials stopped for an informative reason (such as efficacy, futility or safety)  -Ongoing trials that have not surpassed double their anticipated primary completion timeline |
| Reporting:  Systems are in place to ensure timely, complete, and accurate reporting | -A trial is reported if primary outcome results are made available through publication or results deposition on ClinicalTrials.gov |

^1^Column “Conditions for Informativeness” extracted from column1 in eTable 1^1^

Bibliography

1. Zarin DA, Goodman SN, Kimmelman J. Harms From Uninformative Clinical Trials. *JAMA.* 2019;322(9):813-814.
