# Supplementary material for: The proportion of randomized controlled trials that inform clinical practice
Source: eLife. 2022 Aug 17;11:e79491. doi: 10.7554/eLife.79491 (PMC9427100; doi:10.7554/eLife.79491)
Supplement: Supplementary file 12. [file elife-79491-supp12.docx]

**Supplementary File 12 –** **Classification of Reason for Termination**

| **NCT** | **Reason for Termination** | **Outcome** |
| --- | --- | --- |
| NCT00831441 | None Provided | Advanced to next step of assessment |
| NCT00863512 | None Provided | Advanced to next step of assessment |
| NCT00887315 | Accrual; Loss of sponsor | Infeasible |
| NCT00910299 | Futility | Advanced to next step of assessment |
| NCT00932152 | Accrual | Infeasible |
| NCT00965055 | Accrual | Infeasible |
| NCT01041781 | DSMB Recommendation | Advanced to next step of assessment |
| NCT01078272 | Accrual | Infeasible |
| NCT01246011 | Accrual | Infeasible |
| NCT00976677 | None Provided | Advanced to next step of assessment |
| NCT01177592 | Funding | Infeasible |
| NCT01179308 | PI closed study site | Infeasible |
| NCT01197963 | IRB Decision | Advanced to next step of assessment |
| NCT01231750 | Accrual | Infeasible |
| NCT01413750 | Accrual | Infeasible |
