# Supplementary material for: The proportion of randomized controlled trials that inform clinical practice
Source: eLife. 2022 Aug 17;11:e79491. doi: 10.7554/eLife.79491 (PMC9427100; doi:10.7554/eLife.79491)
Supplement: Supplementary file 13. [file elife-79491-supp13.docx]

**Supplementary File 13 –** **Methodology for Publication Search**

The search for publications was independently performed by two authors (NH & HM) and included an evaluation of publication links provided on ClinicalTrials.gov, as well as directed searches on Google Scholar, Scopus and Medline using a combination of the unique trial registration number (NCT number), surname of the principal investigator, indication, intervention, phase and study design. Publication identity was confirmed by comparing trial arms, sample size, intervention details, comparators and sponsor with the registration record. A published abstract was not counted as a full publication. Publication search was repeated in October 2021 by NH & HM for those trials without publications when first assessed.
