# Supplementary material for: The proportion of randomized controlled trials that inform clinical practice
Source: eLife. 2022 Aug 17;11:e79491. doi: 10.7554/eLife.79491 (PMC9427100; doi:10.7554/eLife.79491)
Supplement: Supplementary file 14. [file elife-79491-supp14.docx]

**Supplementary File 14 –** **Systematic Review Citation Search Strategy and Quality Assessment**

Assessment of citation of trial results in high quality systematic reviews (SRs) was independently performed by two authors (NH & HM). This first involved a search for sources that are well known for producing high quality SRs: Cochrane SRs on the Cochrane Database of Systematic Reviews^1^ and Agency for Healthcare Research and Quality (AHRQ) SRs ^2^. If trials were not included in Cochrane or AHRQ reviews, additional SRs for published studies were identified using the Scopus database ^3^ citation analysis search function or via Google Scholar ^4^ for unpublished studies. SRs identified through Scopus or Google Scholar that included trial results in review results were assessed for quality using a modified AMSTAR scoring system:

Operationalization of modified AMSTAR^5,6^ scoring system

| 1. **Was an “a priori” design provided?**   Yes – the authors stated that methods were established prior to conducting the review or provided a link to a registered protocol record  No – the authors stated that there’s no protocol available or no information is provided | □ Yes (1) |
| --- | --- |
|  | □ No (0) |
| 1. **Was there duplicate study selection and data extraction?**   Yes – at least two individuals independently performed study selection and data extraction; the method for reaching consensus in the setting of disagreement was reported  No – only one person performed either study selection or data extraction  Can’t answer – no information about independent study selection and/or data extraction was provided | □ Yes (1) |
|  | □ No (0) |
|  | □ Can't answer (0) |
| 1. **Was a comprehensive literature search performed?**   Yes – at least two electronic sources were searched; keywords or MESH terms were provided  No – only one database was searched; no keywords or MESH terms were provided  Can’t answer – partial or no information reported | □ Yes (1) |
|  | □ No (0) |
|  | □ Can't answer (0) |
| 1. **Was a list of studies (included and excluded) provided?**   Yes - a list of included and excluded studies was provided  No – a list of included and excluded studies was not provided | □ Yes (1) |
|  | □ No (0) |
|  |  |
| 1. **Was the scientific quality of the included studies assessed and documented with a study-specific quality score provided?**   Yes – risk of bias or another quality metric was used and reported  No – no risk of bias or quality metric was used  Can’t answer – the authors state that a quality metric was done, but do not provide additional information | □ Yes (1) |
|  | □ No (0) |
|  | □ Can't answer (0) |

High quality review = score of ≥ 3/5

Trials were deemed to have fulfilled the importance criterion if they were cited in the results of a Cochrane SR, an AHRQ SR, or an SR achieving a modified AMSTAR score of greater or equal to 3 out of 5.

Assessment of citation of trial results in SRs was repeated in October 2021 by NH & HM for those trials without an informative citation when first assessed.

Bibliography

1. Cochrane Database of Systematic Reviews. (<https://www.cochranelibrary.com/cdsr/reviews>)

2. Agency for Healthcare Research and Quality. (<https://effectivehealthcare.ahrq.gov>).

3. Scopus. (<https://www.scopus.com>)

4. Google Scholar. (<https://scholar.google.ca>).

5. Shea BJ, Hamel C, Wells GA, et al. AMSTAR is a reliable and valid measurement tool to assess the methodological quality of systematic reviews. *J Clin Epidemiol.* 2009;62(10):1013-1020.

6. Flodgren G, Eccles MP, Shepperd S, Scott A, Parmelli E, Beyer FR. An overview of reviews evaluating the effectiveness of financial incentives in changing healthcare professional behaviours and patient outcomes. *Cochrane Database Syst Rev.* 2011(7):CD009255.
