# Supplementary material for: The proportion of randomized controlled trials that inform clinical practice
Source: eLife. 2022 Aug 17;11:e79491. doi: 10.7554/eLife.79491 (PMC9427100; doi:10.7554/eLife.79491)
Supplement: Supplementary file 15. [file elife-79491-supp15.docx]

**Supplementary File 15 – Clinical Practice Guideline and Point-of-Care Medical Database Search Strategies and Quality Assessment**

Assessment of citation of trial results in Clinical Practice Guidelines (CPGs) was independently performed by two authors (NH & HM). CPGs were identified via Scopus ^1^ citation analysis for published studies or via Google Scholar ^2^ for unpublished trials. Quality of CPGs was assessed using a modified AGREE II scoring system:

Operationalization of modified AGREE II ^3^ scoring system

| 1. **Were systematic methods used to search for evidence and criteria for selection of evidence clearly described?**   Yes – the authors described electronic databases/sources where search was performed, time periods searched, key terms used; inclusion/exclusion criteria for evidence selection were outlined  No – no description was available/no systematic search for evidence conducted/no criteria for selection of evidence described | □ Yes (1) |
| --- | --- |
|  | □ No (0) |
|  |  |
| 1. **Were the strengths and limitations of the body of evidence clearly described?**   Yes – description of the tools used to assess quality of evidence provided (e.g. GRADE framework) or explicit discussion of the quality of the entire group of included trials provided  No – no evaluation of quality | □ Yes (1) |
|  | □ No (0) |
|  |  |
| 1. **Were the methods for formulating recommendations clearly described?**   Yes – description of the recommendation development process was included (e.g. voting procedures) and level of consensus reached were described  No – no clear description of the process involved in formulating recommendations provided | □ Yes (1) |
|  | □ No (0) |
|  |  |
| 1. **Were the guidelines externally reviewed prior to publication?**   Yes – guidelines were externally reviewed and reviewers were not involved in the guideline development group  No – no external review performed or reviewers not independent of guideline information  Can’t answer – insufficient information to evaluate external review process | □ Yes (1) |
|  | □ No (0) |
|  | □ Can't answer (0) |
| 1. **Were competing interests of guideline developers recorded and addressed?**   Yes – a description of competing interests was provided and their potential impact on guideline development discussed; guideline developers were independent from funding body / funding body did not influence final recommendations  No – no competing interests described, or impact on guideline development not assessed, or unclear if funding body has influenced guideline development | □ Yes (1)  □ No (0) |
|  |  |

High quality review = score of ≥ 3/5

Trials were deemed to have fulfilled criteria for importance if they were cited in the results of a high-quality CPG. The remaining uncited trials were assessed for inclusion in a point-of-care medical database article by two authors (NH & HM). Using disease and intervention keywords, we searched UpToDate^4^ to identify any articles citing the remaining trials.

Assessment of citation of trial results in Clinical Practice Guidelines (CPGs) was repeated in October 2021 by NH & HM for those trials without an informative citation when first assessed.

Bibliography

1. Scopus. (<https://www.scopus.com>)

2. Google Scholar. (<https://scholar.google.ca>).

3. Brouwers MC, Kho ME, Browman GP, et al. AGREE II: advancing guideline development, reporting and evaluation in health care. *CMAJ.* 2010;182(18):E839-842.

4. UpToDate. (<https://www.uptodate.com/home>).
