# Supplementary material for: The proportion of randomized controlled trials that inform clinical practice
Source: eLife. 2022 Aug 17;11:e79491. doi: 10.7554/eLife.79491 (PMC9427100; doi:10.7554/eLife.79491)
Supplement: Supplementary file 16. [file elife-79491-supp16.docx]

**Supplementary File 16 – Operationalization of modified Cochrane Risk of Bias score**

We employed a modified 2011 version of the Cochrane Risk of Bias Assessment Tool (detailed criteria for judging risk of bias provided in Table 8.5d of the Cochrane Handbook for Systematic Reviews of Interventions version 5.1) ^1^. When available, ROB scores were extracted directly from high-quality SRs identified during the assessment of trial importance. When not available, assessment was independently carried out by two authors (NH & HM), with differences resolved by a third (JK). Information from both the primary study publication and the ClinicalTrials.gov registration record were used in our risk of bias assessments. Assessment included the following elements: i) random sequence generation; ii) allocation concealment; iii) blinding of participants and personnel; iv) blinding of outcome assessment; v) incomplete outcome data; and, vi) selective reporting. Trials were deemed of sufficient design quality if all elements were deemed to be “low risk of bias” or if a minority of elements were deemed of “unclear risk” and the remaining were “low risk.” Any “high risk” of bias element equated with poor trial design.

Of the 63 trials assessed for trial design, 36 ROB scores were extracted directly from SRs, the remaining 27 trials were assessed by the study team.

Bibliography

1. The Cochrane Collaboration. Cochrane Handbook for Systematic Reviews of Interventions version 5.1.0. Eds J.P.T. Higgins & S.Green. 2011. <https://training.cochrane.org/handbook/archive/v5.1/>.
