# Supplementary material for: The proportion of randomized controlled trials that inform clinical practice
Source: eLife. 2022 Aug 17;11:e79491. doi: 10.7554/eLife.79491 (PMC9427100; doi:10.7554/eLife.79491)
Supplement: Supplementary file 17. [file elife-79491-supp17.docx]

**Supplementary File 17 –** **Inter-rater Agreement Rates**

| **Category** | **Unweighted Cohen’s Kappa** |
| --- | --- |
| Screening trials for inclusion/exclusion | 0.83 |
| Evaluating trial Feasibility | 0.98 |
| Evaluating trial Reporting | 0.79 |
| Evaluating trial Importance | 0.67 |
| Evaluating trial Design | 0.84 |
