# Supplementary material for: The proportion of randomized controlled trials that inform clinical practice
Source: eLife. 2022 Aug 17;11:e79491. doi: 10.7554/eLife.79491 (PMC9427100; doi:10.7554/eLife.79491)
Supplement: Supplementary file 18. [file elife-79491-supp18.docx]

**Supplementary File 18 –** **Deviations to the Study Protocol**

1. For our feasibility assessment, we first evaluated feasibility of goal patient enrollment and planned date of primary completion based on the first record available on ClinicalTrials.gov (this was independently double-coded). However, we repeated this assessment using the final registration record prior to trial start date as this was felt to provide a better evaluation of feasibility, allowing investigators to adjust enrollment plans and primary outcome timeline prior to trial start. The latter method was single coded and did not produce any change in the results of our feasibility assessment.

2. We performed two additional sensitivity analyses on the primary outcome: i) excluding all trials in the lower quartile of goal patient enrollment; and, ii) excluding all phase 1/2 and phase 2 trials from the assessment.

3. We excluded evaluation of primary outcome integrity from our assessment of trial informativeness, given concerns that there can be scientifically valid reasons for altering a primary outcome. For example, a primary outcome might be changed due to evolving clinical practice or in response to new data from outside the trial.

4. We excluded trials of interventions that were subject to FDA regulations, but were never approved for any indication, or were FDA approved after trial start, but did not have 5 years of follow-up time from 31 October 31 2016 post-approval to allow for enough time for trial results to be included in systematic reviews/clinical practice guidelines/UpToDate.

5. We excluded Indeterminate trials from our cohort, defined as ongoing trials that have not surpassed double the allotted time for primary outcome completion (as first stated in the historical clinicaltrials.gov registration record)
